# Bibliographical Department

**Published:** 1881-08

**Authors:** 


### BIBLIOGRAPHICAL DEPARTMENT.

"Nothing extenuate nor set down aught in malice."

*Index—Catalogue of the Library of the Surgeon-General's Office, United States Army. Authors and Subjects, vol.* 2, *Berlioz-Cholas, Washington, Government Printing Office,* 1881.

Is the title-page to volume second of the great work now going on under the supervision of the Surgeon-General's Department of the U. S. Army, at Washington. What we said last year in commendation of the first volume will apply with equal force and propriety to the second one now before us. Should the succeeding volumes attain to the excellence of the two already issued, it will doubtless be without a peer in the world, when completed. The following from the preface or opening of the volume, will give an epitome of its leading features, and varied contents, viz.:

"War Department Surgeon-General's Office,
*Washington, D. C., June 1st.* 1881.

General Joseph K. Barnes, *Surgeon-General U. S. Army.*

General: I have the honor to present herewith the second volume of this Index-Catalogue of the Library of this office. This volume includes 12,459 author-titles, representing 3,934 volumes and 9,810 pamphlets. It also includes 11,550 subject-titles of separate books and pamphlets, and 37,310 titles of articles in periodicals. I am indebted to Charles Rice, Ph. D., of New York City, for valuable aid in reading the proofs of this volume.

Very respectfully your obedient servant,

J. S. BILLINGS,

Bvt. Lt. Col. and Surgeon U. S. Army."

*A Practical Treatise on Impotence, Sterility and Allied Disorders of the Sexual Organs. By Samuel W. Gross, A.M., M.D.; Lecturer on Venereal and Genito-Urinary Diseases in the Jefferson Medical College of Philadelphia; Surgeon to, and Lecturer on Clinical Surgery in the Jefferson Medical College Hospital and the Philadelphia Hospital;*

*President of the Pathological Society of Philadelphia; Author of a Practical Treatise on Tumors of the Mammary Gland, etc., etc.*

We have received through Messrs. Cushings & Bailey, of Baltimore, a copy of the above brochure from the excellent publishing house of Henry C. Lea's Son & Co., of Philadelphia.

It would be almost superfluous to add that the book is gotten up in excellent style, and that the author has done his part in a most agreeable and pleasant manner. But this would not convey all that should be said of this contribution to our science. It is a scholarly and highly valuable work upon subjects of the greatst importance to every practitioner of medicine, and must command a large and extensive sale.

*The Mid-Continent*, a religious and literary weekly paper, published at Kansas City, Mo., comes to us regularly and is always filled with choice and instructive reading matter. It is liberal and enlightened in its views upon religious subjects, and ably edited by S. B. Bell, D. D., and assistants.

*Tenotony in the Treatment of Congenital Club-Foot. By Ap. Morgan Vance, M. D., New York;* contains an interesting tabular report of fifty-two cases treated by Dr. Vance.

Several college announcements have been received, but too late for notice in this issue.

*The Arkansaw Doctor. A Monthly Journal of Practical Medicine.* Vol. 1, No. 1: June, 1881. L. I. Collins, M. D., editor and proprietor—has just come to hand. It appears that it is the only medical journal in the State of Arkansas, and having a large field it ought to succeed, and we trust it will.

*The Peoria Medical Monthly; A Journal Devoted to Medicine and Surgery.* THOS. M. MCILVAINE, A. M., M. D., *Publisher*, 204 *South Jefferson street, Peoria, Illiuois.*—The June (1881) issue of this neatly gotten up and well-edited journal has reached our table, and is cheerfully placed upon our list of exchanges.

*The Sonlhern Medical Reformer.*—Devoted to Sanative Medicine. Edited by S. F. Salter, M. D., and published at Atlanta, Ga., has also been received and placed among our exchanges. It appears to be conducted with commendable fairness, and will doubtless prove acceptable to the class of practitioners for whose use it seems to be specially intended.

The 16th annual announcement of the N. Y. College of Dentistry—Session of 1881–2. This college is enjoying a vigorous and healthy prosperity. It is the deserved reward of the intelligent and energetic exertions of its very able corps of managers.

